# The Nuremberg Code subverts human health and safety by requiring animal modeling

**DOI:** 10.1186/1472-6939-13-16

**Published:** 2012-07-08

**Authors:** Ray Greek, Annalea Pippus, Lawrence A Hansen

**Affiliations:** 1 Americans For Medical Advancement, 2251 Refugio Rd, Goleta, CA 93117, USA; 2 Department of Neurosciences and Pathology, University of California, San Diego, Mail Code 062, 9500 Gilman Drive (MTF 351), La Jolla, CA, 92093-0624, USA

**Keywords:** Animal, Biological complexity, Ethics, Evolution, Law, Nuremberg code, Species variation

## Abstract

**Background:**

The requirement that animals be used in research and testing in order to protect humans was formalized in the Nuremberg Code and subsequent national and international laws, codes, and declarations.

**Discussion:**

We review the history of these requirements and contrast what was known via science about animal models then with what is known now. We further analyze the predictive value of animal models when used as test subjects for human response to drugs and disease. We explore the use of animals for models in toxicity testing as an example of the problem with using animal models.

**Summary:**

We conclude that the requirements for animal testing found in the Nuremberg Code were based on scientifically outdated principles, compromised by people with a vested interest in animal experimentation, serve no useful function, increase the cost of drug development, and prevent otherwise safe and efficacious drugs and therapies from being implemented.

## Background

Using animals to learn more about life in general and humans in particular dates back to ancient times. In the first century BCE, researchers dissected the optic nerve in living animals, vivisected a pig while it was swallowing colored water in order to evaluate the action, and observed intact beating hearts [1]. Animal experimentation continued with Galen in the first century CE but modern animal use in research and testing dates to Claude Bernard in 19^th^ century France [2].

The notion that testing chemicals on animals could be predictive of human responses and therefore should be legally mandated dates back to the 1930s, when the sulfa drugs were being introduced for infections. Sulfa drugs were some of the first drugs that were shown to be effective against certain bacterial infections, but they were difficult to dissolve in solution. This was a problem, as children usually require a liquid version of a medication because they will not swallow pills. In 1937, one sulfa drug was dissolved in ethylene glycol and subsequently administered to children and adults. The ethylene glycol, which is well-known today as an ingredient in anti-freeze, killed one hundred and seven people. This incident led directly to the enactment of the US *Federal Food, Drug and Cosmetic Act* of 1938 to mandate some animal testing [3].

The Nuremberg Code came out of a trial in post-war Germany in December of 1946, the second of the Nuremberg trials. The first tried 24 Nazis, including Hermann Göring and Rudolf Hess, at the International Military Tribunal for crimes against humanity. This first trial lasted eight months with ultimately seven of the 24 defendants being executed. Some were sentenced in absentia, some were acquitted, some committed suicide or could not be tried for medical reasons, and others were incarcerated [4].

As the first trial progressed it became obvious that more people were responsible than merely the 24 under scrutiny, so a total of 12 more trials were held [5]. These trials were held before US military tribunals, with the sole trier of fact being the United States. Thus the second trial was formally designated *United States of America v. Karl Brandt et al.*, colloquially referred to as the “Doctors’ Trial” or the “Medical Case.” Four judges presided over the eight-month case, hearing 85 witnesses, and viewing 1,471 documents and 11,538 pages of transcript [6]. Twenty out of 23 defendants were physicians. All had been singled out as being responsible for the execution of humans they deemed “unworthy of life” and for experiments conducted on concentration camp prisoners. Experiments contained in the indictments included those pertaining to treatments for persons who had been severely chilled, the effects of various poisons and vaccines, and testing for pharmaceutical treatments for phosphorus burns from incendiary bombs. All of the experiments were performed on unconsenting humans who were inmates of concentration camps [7]. The defendants were charged with and tried for conspiracy to commit war crimes against humanity, war crimes, and crimes against humanity. The tribunal did not convict on the first charge; 15 of the defendants were convicted on counts two and three. Ten of the 23 defendants were further charged with, and found guilty of, membership in a criminal organization (the SS) [7]. The Nuremberg Code also came out of these proceedings, comprising ten ethical ideals that strive to lay the groundwork for ensuring that human rights are respected in human experimentation.

The American Medical Association appointed an advisor to the prosecutor for *US v. Brandt*, Dr Andrew C Ivy [8]. Ivy was a scientist himself and had conducted research similar to what was being discussed at the trial, such as the effects of altitude on pilots [9]. Ivy was also a staunch opponent of those seeking to remove animals from laboratories during the 1930s and 1940s. He was a co-founder of the National Society for Medical Research, an organization that campaigned in favor of animal experiments, and served as its secretary-treasurer for years. It was Ivy who wrote the manuscript the prosecutors used to evaluate the scientific aspects of the charges [10,11]. This manuscript included:

"The experiment to be performed must be so designed and based on the results of animal experimentation and a knowledge of the natural history of the disease under study that the anticipated results will justify the performance of the experiment. . . . The experiment must be conducted . . . on the basis of the results of previous adequate animal experimentation, there is no a priori reason to believe that death or disabling injury will occur, except in such experiments as those on Yellow Fever where the experimenters serve as subjects along with non-scientific personnel."

The American Medical Association quickly adopted Ivy’s rules and this was presented at Nuremberg in such a way as to make it appear that the rules were well established in the US ( [10,11] also see [12]). Ivy’s wording would appear almost verbatim in the ultimate Nuremberg Code. The Declaration of Helsinki, authored by the World Medical Association, was a medical adaption of the Nuremberg Code and came out in 1964. It has been revised six times since then. The Declaration of Helsinki [13] came to supersede the Nuremberg Code as the normative ethical guidance for medical researchers [6]. The Declaration of Helsinki represents an improvement over the Nuremberg Code in the sense that it balances the concerns of individuals against the benefits to the society [14].

Both the Nuremberg Code and the Declaration of Helsinki indicate that animal-based research should be conducted before human experimentation, the former more unequivocally than the latter. Principle 3 of the Nuremberg Code states:

"The experiment should be *so designed and based on the results of animal experimentation* and a knowledge of the natural history of the disease or other problem under study that the anticipated results will justify the performance of the experiment [15]. (Emphasis added.)"

This principle is predicated on the assumption that the animal experimentation will have predictive value for the efficacy of the ultimate experimentation on humans. Ironically, experiments were also conducted on animals in Nazi Germany despite the commonly held position that they were not [16-20]. The following is from a document presented at the Doctor’s Trial (USA v. Karl Brandt, *et al.*) at Nuremberg and is titled “The Blood Picture of the White Mouse in Experimental Infections and Chemotherapy” (spelling per the original document).

"In former works ^1)^ we have reported on the application of hematolytic technique to prove the functional condition of the mesenchyma in artificially infected animals and chemotherapeut treatment. The differential blood picture in normal mice as we as in mice infected with recurring spirochetes and nagana trypanosomes treated with salvarsan and solganol was only briefly discussed and publication at a later date was promised. . . . To cause hyperemia the tails of the mice was dipped for a short while into water of 40^o^C, were severed and a drop . . ."

Principle 12 of the Declaration of Helsinki advises that medical research on humans must be based on animal experimentation as appropriate. Again, the assumption is that animal experimentation will have predictive value for human research or experimentation, thus protecting human rights. As we will see, the opposite is the case: reliance on animal-based research in conducting human experimentation is antithetical to a respect for human rights.

Neither the Nuremberg Code nor the Declaration of Helsinki is legally binding or legally enforceable in its own right. (However, see [21]). They are ethical *guidelines*. Both documents and the principles enshrined in them will be persuasive authority to any domestic court, and indeed an argument can be made that many if not most of the principles are customary law (i.e. international law, binding on all states, that is derived from the customary behaviour of states, indicating a consensus that the behaviour is obligatory). Requiring consent in experiments, for example, may be considered a principle of customary international law, and states may have recourse at the International Court of Justice if this principle is violated. However, international codes and declarations gain tangible lawful force for individuals when they are adopted into domestic laws. The USA Protection of Human Subjects [22] reads as follows:

§46.118 Applications and proposals lacking definite plans for involvement of human subjects.

Certain types of applications for grants, cooperative agreements, or contracts are submitted to departments or agencies with the knowledge that subjects may be involved within the period of support, but definite plans would not normally be set forth in the application or proposal. These include activities such as institutional type grants when selection of specific projects is the institution's responsibility; research training grants in which the activities involving subjects remain to be selected; and projects in which human subjects' involvement will depend upon completion of instruments, prior animal studies, or purification of compounds.

§46.204 Research involving pregnant women or fetuses.

Pregnant women or fetuses may be involved in research if all of the following conditions are met:

(a) Where scientifically appropriate, preclinical studies, *including studies on pregnant animals*, and clinical studies, including studies on nonpregnant women, have been conducted and provide data for assessing potential risks to pregnant women and fetuses . . .(Emphasis added.)

In Canada, s. 30 of the *Food and Drugs Act*[22] provides that the Governor in Council may make regulations for carrying the purposes and provisions of the Act into effect. The consequent *Food and Drugs Regulations*[23] reference animal-based research in at least three separate provisions. Provision C.08.002.01 provides that a manufacturer of a new drug may file an extraordinary use new drug submission if the new drug is intended for:

(i) emergency use in situations where persons have been exposed to a chemical, biological, radiological or nuclear substance and action is required to treat, mitigate or prevent a life-threatening or other serious disease, disorder or abnormal physical state, or its symptoms, that results, or is likely to result, from that exposure, or

(ii) preventative use in persons who are at risk of exposure to a chemical, biological, radiological or nuclear substance that is potentially lethal or permanently disabling; and

However, s. C.08.002.01(2)(iv) requires that the submission for extraordinary use new drugs contains:

(iv) detailed reports of studies, in an animal species that is expected to react with a response that is predictive for humans, establishing the safety of the new drug, and providing substantial evidence of its effect, when used for the purpose and under the conditions of use recommended,

(v) information confirming that the end point of animal studies is clearly related to the desired benefit in humans,

(vi) information demonstrating that there is a sufficient understanding of the pharmacokinetics and pharmacodynamics of the new drug in animals and in humans to enable inferences to be drawn in respect of humans so as to allow for the selection of an effective dose in humans, . . .

Other provisions in the *Regulations* suggest an assumption that animal-based research has predictive value for humans, although interestingly, none—other than the foregoing—*require* the results of animal-based research. Rather, it is indicated that when animal-based research exists its results should be included in applications for drug authorization. In other words, with the exception of extraordinary use drugs, animal-based research does not appear to be mandated under the *Regulations*.

For example, provision C.05.005(e) provides that an application to sell or import a drug for a clinical trial involving human subjects shall contain an investigator’s brochure containing a variety of information, including

(ii) the pharmacological aspects of the drug, including its metabolites *in all animal species tested*,

(iii) the pharmacokinetics of the drug and the drug metabolism, including the biological transformation of the drug *in all animal species tested*,

(iv) any toxicological effects *in any animal species tested* under a single dose study, a repeated dose study or a special study in respect of the drug,

(v) any results of carcinogenicity studies in *any animal species tested* in respect of the drug, . . . [Emphasis added.]

Only when animal species have been tested should that information be included in the application. If the pharmacological aspects, pharmacokinetics, toxicological aspects, and carcinogenicity of the drug can be demonstrated using non-animal models, this is sufficient.

Regulations are law that is not enacted by the legislature, but rather is created by those to whom authority has been delegated under the governing act. They can be amended by the delegated authority. The *Food and Drugs Act* allows the Governor in Council, whose decision-making is, in practice, undertaken by cabinet, to make regulations for that act. Moreover, all federal regulation-making in Canada is governed by the *Statutory Instruments Act*[24]. Section 19.1(1) of the *SIA* provides that a legislative committee may revoke all or part of any regulations. The development, implementation, evaluation, and review of regulations are further governed by the *Cabinet Directive on Streamlining Regulation*. Among other things, this policy document requires the federal government to protect and advance the public interest in health, to make decisions based on evidence and the best available science, and to be efficient and effective by demonstrating tangible results for humans. If animal-based research does not advance the public interest, is not scientifically valid, and/or does not demonstrate tangible results for humans, then it cannot be required under federal regulations such as the *Food and Drug Regulations*.

In 2009, animal testing to fulfill regulatory requirements, category PAU 3, accounted for 66% (96,211 animals) of Canadian experiments known to “cause pain near, at, or above the pain tolerance threshold of unanesthetized, conscious animals” [25].

Similarly, the US Food and Drug regulations stipulate that results from animal-based research should be included in applications if it has been conducted (e.g. s. 314.50), but the plain meaning of the text is that it is not mandated. For example, Part 314, Subpart I of the Food and Drug Regulations set out standards for the “approval of new drugs when human efficacy studies are not ethical or feasible.” In such circumstances, the FDA will accept “adequate and well-controlled animal studies when the results of those animal studies establish that the drug product is reasonably likely to produce clinical benefit in humans.” The presumption underlying these regulations is that animal studies have predictive value for humans. The FDA does not require proof of efficacy in animal models while they do in practice mandate toxicity testing in animals. This should be interpreted in light of the fact that what the FDA *requires* differs from what the FDA *accepts* and in some cases this is a distinction without a difference. If efficacy has not been demonstrated in an animal model, the investigational new drug approval process can be far more complicated and difficult. In addition, there is variability in the approval process.

Nevertheless, the US Food and Drug Administration (FDA) states that it requires animal testing on a rodent and nonrodent species in order to determine toxicity in humans, the dose to administer to humans taking a new drug for the first time, in order to establish a margin of safety, and for monitoring purposes during clinical trials [26].

## Discussion

### Science - theory

At the time of the Nuremberg trials, medical science was very different than it is now. The structure of DNA had not been elucidated, scientists thought the poliovirus entered via the nose (it enters through the gut) [27], the notion of a *magic bullet* (that for every disease, or at least every infectious disease, a chemical existed that could interact with the single site causing the malady and thus cure the disease without harming the rest of the body) via Ehrlich and Salvarsan [28] was foremost in the minds of drug developers, the modern synthesis in evolution was brand new [29], and animals and humans seemed to be more or less the same except for humans having a soul [2,30,31]. There were no organ transplants, infectious diseases were still a major killer in the developed world, the fields of cognitive ethology and animal cognition were unheard of, and differences between ethnic groups [32-38] and sexes [39-43] in terms of disease and drug reactions had not yet been discovered. Physics was just beginning to cast off the shackles of determinism and reductionism but chaos and complexity theory was still on the horizon. It was a different world. People in the 1940s are to be excused for thinking that animals and humans would react more or less the same to drugs and disease. We will now bring the reader into the current scientific environment as it relates to our topic [30,44-49].

Two major advances in science, as it relates to our topic, have occurred since the Nuremberg trials. First, the field of evolutionary biology continued to develop. The new division of evolutionary biology known as evolutionary developmental biology, or evo devo, is one example of the important advances in the field of evolution. Evo devo arguably began in 1978, when Lewis [50] published his findings on the anterior–posterior layout of the fruit fly, *Drosophila*. In 1984, the homeobox genes were discovered by McGinnis et al. [51]. The homeobox genes are responsible for the body plan of “bilaterian” organisms. Bilaterians, of which humans are an example, are symmetrical around two axes. The homeobox genes are responsible for the way the body is configured: the arms here, the thorax there and so on [52]. The homeobox are active in early embryogenesis, organizing the cell and anterior–posterior body layout [53]. While there are differences among species—for example, there are nine homeobox genes in flies contrasted with thirty-nine in mammals—the overall use of the homeobox is the same. Discoveries such as the homeobox allowed scientists to appreciate the fact that mammals, and animals in general, have much in common in terms of their genetic composition. The differences among species were not to be completely explained by different species having different genes.

The concepts learned from evo devo and evolutionary biology in general tie in closely with discoveries from the Human Genome Project (HGP) [54,55] and other spin-off projects. Prior to the HGP, scientists thought the number of genes was proportional to the complexity of the organism. The number of genes in some organisms was known or approximated; therefore, the scientists involved in the HGP were looking for an estimated 100,000+ genes in humans. As the project advanced, it became clear that humans had nowhere near this many genes. This was perplexing.

**Figure 1:**
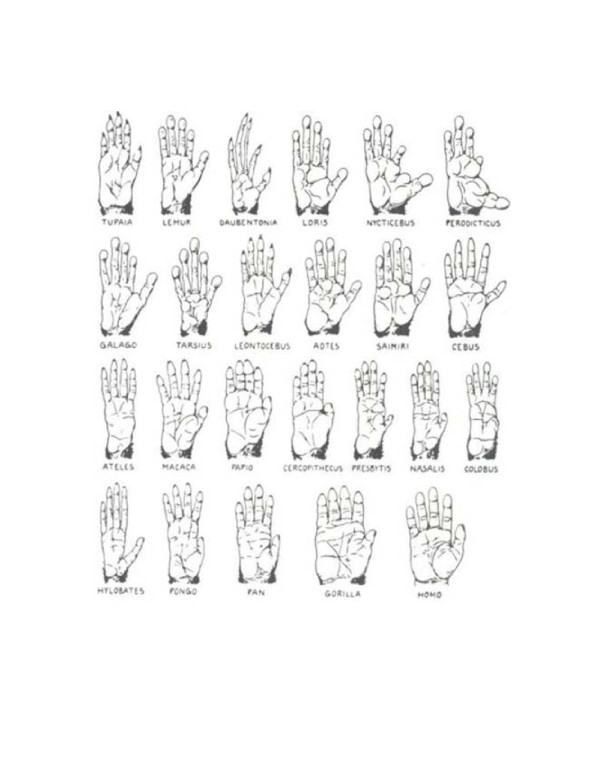
Thumb position and finger length among primates.

Because of evo devo, the HGP and its spinoffs, and speculation by King and Wilson [56] in the 1970s, scientists now know the following. All mammals have more or less the same genes. Some species have a few genes that other species do not have, but one could more or less build any mammal using the genes from another. The differences among species lie, in large part, in the *regulation* and *expression* of the same genes. The genes that build the body are known as structural genes, while the genes that turn the structural gene on and off are called regulatory genes. Think of your genetic composition as the keys on a piano. Every piano has the same keys (structural genes). But each piano can be played so as to produce a variety of tunes. The reason for this is that the structure of the piano allows for keys to be pressed at various intervals and in various combinations. The sheet music dictates when and how to press the keys. Likewise, the regulatory genes (the sheet music) tell the structural genes (the keys) when to be active (expressed) and for how long. For example, humans and mice both have the gene that allows mice to grow a tail. In humans, this gene is not activated during embryogenesis, hence humans have no tail. (Evidence for this is found in the fact that, very rarely, this gene will be turned on in humans and the baby will be born with a tail.) Figure 1, from the early 20^th^ century primatologist Adolph H Schultz, shows the position of the thumb and length of the fingers for various primates. These traits can be determined by how long a gene or set of genes is activated for thus allowing the thumb position to migrate down the hand or the fingers to lengthen.

There are other differences among species and almost all are related to evolution. Table 1[57-59] shows some differences in the composition of enzymes that metabolize drugs. Different enzymes metabolize different drugs, metabolize the same drugs at different rates, and form different metabolites, all of which influence toxicity and dosing. There are also differences in how many copies of a drug-metabolizing gene various animals have. If species A has 10 copies and species B has one copy, then species A might metabolize a drug 10 times faster than species B. This also has significance for dosing and for toxicity. For example, trastuzumab (Herceptin), an anti-breast cancer drug, is prescribed for women who carry multiple copies of, or overexpress, the gene *HER-2/neu*[60].

**Table 1:** CYP enzymes in humans, rat, mouse, dog and monkey

**CYP 450 enzyme**						
**Family**	**Subfamily**	**Gene**				
		**Human**	**Monkey**	**Dog**	**Rat**	**Mouse**
1	A	1, 2	1, 2	1, 2	1, 2	1, 2
2	A	6, 7, 13	23, 24	13, 25	1, 2, 3	4, 5, 12, 22
2	B	6	17	11	1-3, 8, 12, 15, 21	9, 10, 13, 19, 23
2	C	8, 9, 18, 19	20, 74, 75, 76	21, 41	6, 7, 11–13, 22–24, 46	29, 37–40, 44, 50
2	D	6	1, 7, 42	15	1-5, 18	9-13, 22, 26, 34, 40
2	E	1	1	1	1	1
3	A	4, 5, 7, 43	6, 8, 64, 66	12, 26	1, 2, 9, 18, 23, 62	11, 13, 16, 25, 41, 44, 57, 59

Cytochrome P450 (CYP) is a superfamily of enzymes and are the major drug-metabolizing enzymes.

CYP enzymes are categorized as CYP letter_number_letter. The first number is the gene family, the letter is the subfamily, and the final number is the individual gene. Some subfamilies have enzymes from several different genes. (Adopted and based on data from [58-60]).

Species, and even individual humans, can differ in genetic composition. For example, there may be differences in

• The presence (or absence) of certain genes.

The presence (or absence) of certain alleles.

• The background genes and modifier genes that influence the genes being perturbed by drugs or disease.

• The regulation and expression of genes.

Gene networks.

• Alternative splicing, which allows one gene to form or be part of forming many different proteins.

• Proteins and protein–protein interactions.

Gene–protein interactions.

• Old genes evolving to perform new functions.

• Horizontal gene transfer (HGT). HGT occurs when genes from one organism are incorporated into another organism without the recipient organisms being the offspring of the donor. For example, resistance to anti-bacterial drugs can occur through HGT.

• Epigenetics. Epigenetics is the relatively new field that studies changes in gene expression that can be inherited and that occur without changing the underlying DNA sequence. For example, because of environmental influences, a regulatory gene may be changed such that it is turned on or off thus allowing a disease to manifest.

• The presence of gene and chromosomal mutations such as single nucleotide polymorphisms (SNPs), copy number variants (CNVs), duplications, inversions, deletions, and insertions.

n response to a perturbation to the system, such as a drug or disease, even one of the above differences can result in life or death consequences. Furthermore, *convergent evolution* can result in the same trait being present but being mediated by very different pathways in different species. Different molecules can also perform the same function. All of these types of differences are present in every species.

There are, of course, similarities among species. Some of these similarities are referred to as *conserved processes*, which are basic functions of a cell that have been present since early evolutionary times. The homeobox, described above, is an example of a conserved process. Conserved processes occur in living complex systems that have differences like those outlined above. These differences result in the conserved process being influenced by various factors that are unique to each species and even each individual within each species. Importantly, we understand how modifications in the genome, like those mentioned above, have resulted in the evolution of different body types and indeed different species [52,61-63]. Therefore, even when animals and humans share genes and traits, they will most likely still react differently to diseases and drugs.

The second major change in science that is relevant to our discussion is the development of chaos and complexity theories, replacing outmoded deterministic paradigms. For centuries, physics, and science in general, saw the world through Newton’s eyes. Newtonian physics is closely connected to reductionism and determinism. Reductionism maintains that everything can be reduced to its component parts, those parts examined and understood, and then the whole explained based on it being the sum of the parts. Determinism means that once for certain systems, once the initial conditions are known only one outcome is possible. Reductionism and determinism lead to a very linear process with A leading to B leading to C and so on. The Newtonian physics of inclined planes, velocities, forces, a point representing an object, and so forth explores simple systems amenable to reductionism and determinism. The early 20^th^ century saw advances in science that challenged reductionism and determinism. For instance, relativity and quantum mechanics revolutions in physics could not be explained by reductionism. Later in the 20^th^ century chaos and complexity science would be developed, thus changing the way reductionism and determinism were viewed by all of science.

Reductionism worked very well for science and still has a role to play. But some systems are not the simple systems that conform so well to study by reductionism. Some systems are *complex systems* and have rules and characteristics of their own. Complex systems are more than merely a sum of their component parts. Complexity is related to chaos theory. Chaos is perhaps best understood by examining the original experiments performed by Lorenz in 1961. While running weather simulations on a computer, Lorenz shorted a number in an equation from six decimal places to three. When he re-ran the program he found the results were very different from the original. Translating from computer-speak, what he found was that where it had been sunny on day 15, it now rained. Because of the extremely small change in the initial conditions of the program, the outcome was essentially the opposite from the original. This very small change in initial conditions is what phrases like “a butterfly flaps its wings in China and causes a tornado in Kansas” are referring to. Seemingly unimportant differences between two situations or systems can translate into major differences in outcomes. For example, you may eat chocolate but it can kill your dog. The reason for this is the fact that dogs lack the enzyme, or have the enzyme but only in very small quantities, that metabolizes a potentially toxic ingredient in chocolate known as theobromine. Something as simple as the presence or absence of an enzyme can have fatal consequences.

Lorenz’s computer experiment, along with work done by other scientists including Poincaré, gave rise to chaos theory and complexity theory. A major difference between chaotic systems and complex systems is that chaotic systems are deterministic. Given enough computer power and knowledge of the system, outcomes could be predicted. This is not the case with complex systems because they exhibit, among other things, *emergent* properties. Emergence is the presence, in a system, of new properties that could not have been predicted even with total knowledge of the component parts from which the emergent property arose. Financial markets, the behavior of ant colonies, cells, and living organisms are examples of complex systems whereas the weather and the red spot on Jupiter are examples of chaotic systems.

Complex systems, including humans and other animals, have the following characteristics:

1. The whole is greater than the sum of the parts. This is, in part, because of emergent properties. Because complex systems exhibit the characteristics of emergence and the whole being greater than the sum of its parts, they cannot be completely described via reductionism.

2. Different levels of organization exist and a perturbation to the system as a whole may affect each level differently.

3. There are a large number of components or parts and these can combine to form modules that interact with each other and the environment. Feedback loops also exist among the parts and modules.

4. The system displays robustness, meaning it is resistant to change, and redundancy, meaning that the loss of one part may be compensated for by another part.

5. Complex systems are best described by differential equations and are examples of nonlinearity. Nonlinearity means that a small perturbation may have no effect on the system or a very large effect. Cause does not give rise to effect in the linear way it does in a simple system.

6. The particular manifestations of complex systems and chaotic systems are both determined in part or in whole by initial conditions. For example, changing or deleting just one gene in a living complex system might result in death or in no noticeable change whatsoever. This has important implications as studies have demonstrated that deleting a gene in a mouse may result in the death of one strain but not another. Similarly, a gene may be required for human development but not the development of mice or other animals.

Humans and animals are living complex systems that have different evolutionary trajectories. Therefore, animals and humans have very different initial conditions in the form of the genetic differences listed above. It follows that one species may respond to perturbations such as drugs and disease in a manner that cannot be predicted based the response of a different species. Moreover, all of the characteristics of a complex system, and the differences between complex systems that have occurred because of evolution, have a major impact on inter-species extrapolation. This was not appreciated during the era of the Nuremberg trials. Predicting outcomes within a complex system is problematic; predicting an outcome for one complex system based on the outcome from another is virtually impossible. Nevertheless, this is exactly what scientists are attempting to do when they test a drug on a mouse or monkey in an attempt to ascertain what it will do to a human.

With the above in mind, we will now examine the results—the empirical evidence—of attempting to predict human outcomes by using animals in toxicity testing.

### Science – empirical evidence

Paracelsus [64] pointed in the 16^th^ century that it is the dose of a chemical that determines whether it is toxic. All chemicals, even air and water, can be toxic if administered in the right dose and all chemicals have a dose at which no adverse effects are observed. Too much oxygen, e.g., breathing 100% oxygen for several days, will damage the lungs. Too much water will cause seizures due to electrolyte imbalances. Conversely, one molecule of arsenic is not going to kill you. All medications can likewise cause harm if given at too high of a dose. So how can we determine when a chemical will be toxic and to whom it will be toxic? Goldstein and Henifin have identified three problems in evaluating drugs in development. First, they explain that toxicity is determined in part by other properties of the drug such as absorption, distribution, metabolism, and elimination [65]. Drugs are evaluated for Absorption (A), distribution (D), metabolism (M), and excretion or elimination (E), collectively referred to as ADMET, by various methods including animal models. The second problem is that these properties vary considerably among humans. Indeed, humans vary so much in our response to chemicals that the majority of drugs are only an option for a minority of patients. Some people will tolerate the drug, but the drug will not be effective for them. Others will not tolerate the drug, because of toxicities, even though the drug would have been effective. According to Roses: “The vast majority of drugs - more than 90% - only work in 30 or 50% of the people” [66]. Physicians have long known that there were differences in disease susceptibility and drug response among ethnic groups, [32-38] between the sexes, [39-43] and even between monozygotic twins [67-70]. These facts alone should give us pause when considering using animals as models for humans. For which humans are we assuming the animal will predict a response?

The problem of intra-human variation has led to a new area in medicine called *personalized medicine*. Personalized medicine is the concept of matching disease susceptibility and drug response to genotype in order to individualize patient care. This is already occurring with some drugs and diseases [71-81]. Considering the fact that there is so much intra-species variation in response to perturbations like drugs and disease, it is highly unlikely that attempting to derive toxicity and efficacy data from another species will be productive.

This leads us to problem number three, which as Goldstein and Henifin explain is that extrapolation across species is unreliable [65]. Despite the aforementioned problems, Goldstein and Henifin assure society that: “the toxic responses in laboratory animals are *useful predictors* of toxic responses in humans” [65]. (Emphasis added.) Unfortunately, this sentiment is common in the scientific literature. After explaining why animal models should not be predictive, the authors usually feel obliged to place a disclaimer at the end of the article saying society should continue to support animal-based research. (For more on this see [46].) Such obviously conflicting statements contribute much to society’s confusion about the value of animal models.

The presumed ability of animals to predict human response lays at the foundation of statements like the above and the Nuremberg Code. It is merely assumed that animal models can predict human response. Moreover, scientists and advocates for using animal models actively proclaim that animal models are predictive. Consider the following from Gad, writing in *Animal Models in Toxicology*:

"Biomedical sciences’ use of animals as models [is to] help understand and *predict* responses in humans, in toxicology and pharmacology . . . by and large animals have worked exceptionally well as *predictive* models for humans . . . Animals have been used as models for centuries to *predict* what chemicals and environmental factors would do to humans…. The use of animals as *predictors* of potential ill effects has grown since that time . . . If we correctly identify toxic agents (using animals and other *predictive* model systems) in advance of a product or agent being introduced into the marketplace or environment, generally it will not be introduced . . . The use of thalidomide, a sedative-hypnotic agent, led to some 10,000 deformed children being born in Europe. This in turn led directly to the 1962 revision of the Food, Drug and Cosmetic Act, requiring more stringent testing. *Current testing procedures (or even those at the time in the United States, where the drug was never approved for human use) would have identified the hazard and prevented this tragedy*[82]. (Emphasis added.)

While the above is not subtle, neither is it unique. Hau states in the *Handbook of Laboratory Animal Science*:

"A third important group of animal models is employed as *predictive* models. These models are used with the aim of discovering and quantifying the impact of a treatment, whether this is to cure a disease or to assess toxicity of a chemical compound [83]."

Michael F. Jacobson, executive director of the *Center for Science in the Public Interest* noted in 2008: “We must test animals to determine whether a substance causes cancer” [84]. Similarly, Huff *et al.* observe: “Chemical carcinogenesis bioassays in animals have long been recognized and accepted as valid predictors of potential cancer hazards to humans” [85]. There are many more examples, as the predictivity of animal models is currently a widely accepted paradigm in science.

Before we examine the actual empirical evidence, we need to explain how a practice, method, or modality is assessed in terms of whether or not it qualifies as predictive. Any practice can be assessed. Whether the practice is a medical test, a medical therapy, or a method that lies outside of medical science—for example, how well a drug-sniffing dog performs his job—the modality can be assessed in the following manner. One calculates the sensitivity, specificity, positive predictive value (PPV), and negative predictive value (NPV) of the practice by using the values and calculations in Table 2. PPV refers to the rate at which some intervention correctly predicts the *existence* of some factor, whereas negative predictive value refers to the rate at which some intervention correctly predicts the *inexistence* of some factor. In medical science, a practice is not predictive unless it has a very high PPV and/or NPV. (Some tests are used only to test whether the trait in question is *not* present, hence require only a high NPV.) Values around 0.9 or above on a scale of 0 to 1 (in other words, highly positively or negatively predictive) are needed in medical science and medical practice.

**Table 2:** Binary classification test

		**Gold Standard**	
		GS+	GS-
Test	T+	TP	FP
	T-	FN	TN
Sensitivity = TP/(TP + FN)			
Specificity = TN/(FP + TN)			
Positive Predictive Value = TP/(TP + FP)			
Negative Predictive Value = TN/(FN + TN)			

Allows calculations for determining how well a test or practice compares with reality or the gold standard.

T- = Test negative.

T + = Test positive.

FP = False positive.

TP = True positive.

FN = False negative.

TN = True negative.

GS- = Gold standard negative.

GS + = Gold standard positive.

One final note before we examine actual test results. Some people misinterpret a PPV of 0.5 or 50% as meaning that the test allows scientists to abandon 50% of the drugs that would have injured humans or that drugs as a whole are 50% safer than they would otherwise have been. Such interpretations are incorrect. A PPV of 0.5 means that the probability that any given toxic reaction that was seen in animal will be seen in a human is 50%. That is equivalent to tossing a coin in order to determine whether to proceed with development. PPVs of this value are not even remotely useful in medical science.

We will now examine the empirical evidence in order to determine whether animal models can, in fact, be used to predict human response. We will focus on the use of animals in drug development.

In 1962, Litchfield [86] studied rats, dogs, and humans in order to evaluate responses to six drugs. Only side effects that could be studied in animals were calculated. 89 physical signs were evaluated in the three species. The results are in Table 3.

**Table 3:** Three-way toxicity test results

Man	Toxic effects found in man	53
	Toxic effects found in man only	23
Rat	Toxic effects also found in man	18
	Toxic effects not found in man	19
Dog	Toxic effects also found in man	29
	Toxic effects not found in man	24
Rat	19 false positives 35 false negatives Sn = 18/(18 + 35) = 34% PPV = 18/(18 + 19) = 49%	
Dog	24 false positives 24 false negatives Sn = 29/(29 + 24) = 55% PPV = 29/(29 + 24) = 55%.	

The rat gave a PPV of 0.49 while the dog gave a PPV of 0.55. A PPV around 0.5 is not sufficient to qualify a modality as predictive. It is what one would expect from tossing a coin. Medical science demands values around 0.9 or higher. As we will see, the results from using animal models to predict human response have not changed over the decades. Animal-based testing and research is not resulting in better predictive values for humans.

A specific example from the 1960s is Isuprel (isoproterenol), which is a medication used to treat asthma. It proved devastatingly toxic for humans in the amounts recommended based on animal studies. Thirty five hundred asthmatics died in Great Britain alone. It is still difficult to reproduce these results in animals [87-93]. With respect to the futility of animal models for testing isoproterenol, scientists commented that “[i]ntensive toxicological studies with rats, guinea pigs, dogs and monkeys at dosage levels far in excess of current commercial metered dose vials have not elicited similar results” [89].

In 1978, Fletcher reported on 45 recently developed new drugs, estimating that only up to 25% of the toxic effects observed in animal studies were expected to occur in humans [94]. Since the raw data from this study is not available, it is impossible to calculate PPV and NPV. However, data from animal models that leads to a mere 25% of observed toxic effects being seen in humans denotes the modality is not predictive. Likewise, Heywood in 1981 reported the results of toxicity testing in rodents and non-rodents for 50 compounds and found a 20% correlation. He concluded that inter-species extrapolation was unrealistic [95]. Heywood described a follow-up study in 1983 in which dogs and rats were both studied for toxicity and correlations of less than 50% were demonstrated [96]. Note that a correlation of 50% is not the same as PPV or NPV of 50%. As a measurement tool, correlation is more akin to sensitivity (see Table 2). A high sensitivity does not equal a high PPV. Regardless, even if the PPV had been 50%, this would mean that, per Table 2, the practice is not predictive for medical science.

David Salsburg of Pfizer addressed carcinogen testing in 1983. He reported on the results of testing 170 compounds. He observed that the animal-based tests lacked specificity and sensitivity. He stated that the lifetime feeding study in rodents had less probability of finding known human carcinogens than tossing a coin [97]. Garattini reviewed the literature in 1985, in addition to reporting that his results from testing caffeine in mice, rats, rabbits, monkeys and humans varied widely. He concluded that even in the presence of equal concentrations of metabolites, the effects vary among species because of differing sensitivities and hence extrapolations among species would be specious [98].

In 1990, Heywood reported on drugs that proved so toxic they were withdrawn from clinical trials or the market in the UK. Heywood noted that animal data correlated with human data for severe adverse drug reactions 14% of the time. He estimated that the animal correlation rate in general for adverse reactions that occurred in humans ranged between five and 25% [ [99] p57-67]. Obviously any value in this range, even if it were a PPV as opposed to correlation, is not sufficient for a test to qualify as predictive. Moreover, the Heywood report draws attention to another flaw in such studies. The data from animals was taken as a collective, meaning that any animal that corresponded the same as humans was counted as a positive. In order to conduct a true analysis, the species would have to be categorized individually and each result counted just for that species. Species could then be combined, for example a side effect that occurred in either dog or monkey could be counted as such, but that would have to be made clear and the negatives would also count in the calculations described in Table 2. This would give a much smaller predictive value than a 14% correlation portrays. This is why in many animal-based studies, for example Olson 2000 [100], report correlation or concordance among many species as if it were one value—one value for the animal model *per se*—instead of values for each species or a combination of species.

A similar study examined six drugs, the side effects of which were already known in humans. The study found that at least one species demonstrated correlations for 22 side effects, but incorrectly identified 48 side effects that did not occur in humans, while missing 20 side effects that did occur in humans. This translates to a PPV of 0.31 [ [101] p73].

In one small series, also reported in 1990, that studied drugs cancelled during clinical trials because of toxicity, it was found that in 16 out of 24 (67%) of the cases, the toxicity had no correlation in animals [ [102] p49–56]. A 1994 study revealed that only six of 114 clinical toxicities had animal correlates [ [103] p57-67] and there are many more examples of this theme in the literature [104] [ [105] p67-74] [106-110]. While the data does not allow the calculations in Table 2 to be made, obviously these numbers fall far short of qualifying as a predictive medical test. In 1995, Lin compared pharmacologically important parameters in different species and pointed out that many examples of animal models *predicting* human response were in fact *retrospective* and hence not predictive at all [111].

 Many of the most commonly used animal-based tests, like the Draize test (in which a substance is placed in a restrained animal’s eye and the effects observed) and the LD50 (in which a substance is administered to a group of animals and the dose at which half of them die is recorded), were never considered predictive for humans [112,113]. Dawson et al. studied the role of pesticides in suicide attempts and suicides in Sri Lanka, where pesticides are commonly used for suicide because they are inexpensive and widely available. They found that the WHO toxicity ranking, based on LD50 in rats, did not correlate to toxicity in humans. In other words, the accepted toxicity rating for these pesticides—and any consequent policy—was false because it was based on rat studies that did not correctly predict toxicity in humans. The herbicide Paraquat, for example, was far more lethal in humans than would have been anticipated by the LD50 [114,115].

**Figure 2:**
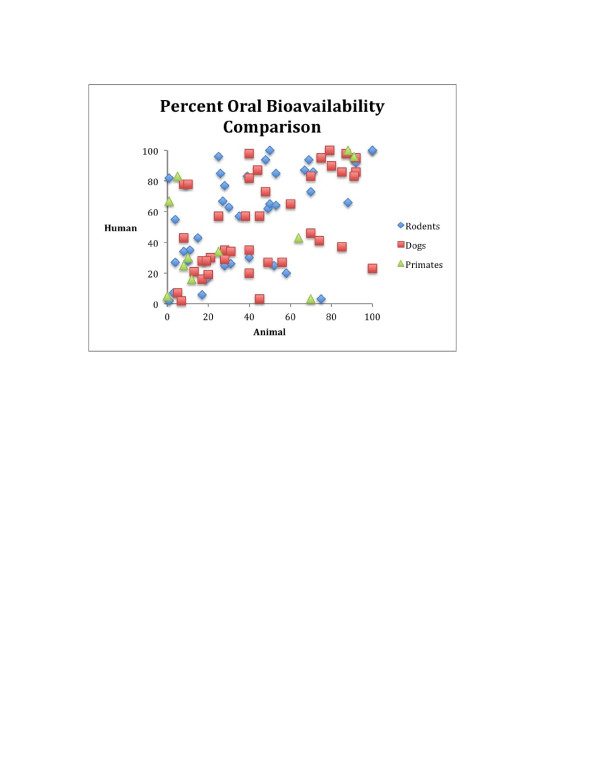
Variation in bioavailability among species.

The results from toxicity testing are not unique in lacking predictive value. In 1989, Sietsema summarized the comparative pharmacology literature concerning the bioavailability of over 400 drugs (see Figure 2, created by authors using data from Sietsema). This graph exhibits a pattern called a scattergram, meaning that the pattern is what one would expect from a shotgun blast—no correlation whatsoever and clearly no predictive capacity. While conceding that relative comparisons might be made between species, Sietsema concluded that “[i]n general, absolute oral bioavailability does not correlate well between species” [116].

By the 2000s, the pharmaceutical industry was acknowledging the inability of animal models to predict human response [117]. Browne and Taylor noted in 2002 that greater than 50% of drugs that fail in clinical trials do so because of efficacy or toxicity, both of which rely on animal models. They also noted that before being recalled, a number of drugs released between 1997 and 1998 were given to approximately 20 million patients in the US. Thus 20 million people were exposed to potentially life-threatening prescription drugs [118]. Sankar, in 2005, pointed out a less appreciated but no less troubling fact about animal testing, observing that many drugs that would have proven beneficial to humans had probably failed some aspect of animal testing and thus patients had lost the use of those drug [110]. The US National Cancer Institute has stated that efficacious anti-cancer drugs have been lost secondary to animal studies [119].

The number one reason for canceling drugs in clinical trials and one of the top reasons for withdrawal of drugs is hepatotoxicity, which is chemical-induced liver damage [117,120-122]. Fourches *et al.* analyzed MEDLINE abstracts for 1061 compounds known to cause hepatotoxicity and discovered that the concordance between species was only 39-44% [123]. (Concordance, like correlation, simply means that an effect was reproduced in an animal species not that the animal had that percentage for predictive values. Also, as we noted in the Heywood report, any instance of the same effect in any species was counted as a positive.) Many studies have been published outlining the countless differences between species that impact on predicting toxicity [124-139].

First-in-human (FIH) studies are the first clinical trials in which a drug is tested in humans after being tested in animals. FIH dosage estimates from animal studies have not been accurately predicted. One of the most notable failures was the first time the drug TGN1412 was administered. TGN1412 was a CD28 superagonist antibody designed for patients suffering from autoimmune diseases. Despite a dose of less than 1/500^th^ of that indicated by the animal species the most sensitive to TGN1412, six human volunteers ended up in the intensive care unit with acute, profound toxicity from TGN1412 [140,141]. Other FIM trials have resulted in deaths [142,143]. Chapman [142] states that: “A major factor complicating risk analysis in FIH trials is the difficulty of making accurate predictions from preclinical laboratory research on human tissues and animal studies of the likely effect of the investigational agent on humans.” Chapman refers to the Horstmann study [144] that examined Phase I trials for cancer therapy and found that found that “15 percent of subjects in trials of single chemotherapy agents experienced serious but nonfatal toxic events.” Fifty-eight deaths were also discovered in this study.

Chapman goes on to describe the various ways this inability to make accurate predictions can lead to harm. First, animal models can fail to predict an adverse effect that does occur in humans. Second, a drug may be efficacious in an animal model but not in humans, thus exposing humans to the risks associated with the drug despite having no possible good come from taking the drug. Moreover, pharmaceutical companies and consumers bear the vast expense of the failed drug development. Finally, animal models may demonstrate adverse effects that humans would not have suffered, and an otherwise good drug will be withdrawn from development. Again, both pharmaceutical companies and, more importantly, patients are harmed by this.

Scientists try to match the animal species most likely to react like humans to the drug being tested. This is fanciful, however, as animals and humans are complex systems, and much that is needed to be known in order to determine how humans will respond cannot be known until after the drug is tested on them. Hence it is impossible to know which animal species will resemble humans until after the fact. Moreover, as we pointed out, the profound variation among humans also limits the predictive possibilities for animal models. Lavery states that, for these reasons, animal models are poor predictors for human response [145] and unsuitable for demonstrating “proof of principle” [146]. Giri and Bader summarize the situation saying: “Clearly, drug testing on animals is unrealistic and causes unforeseen reactions in human clinical trials” [117].

This lack of predictive ability in drug development extends to animal models of disease. Enna and Williams have identified as a major hurdle in translational medicine—the branch of medicine that translates knowledge from divergent areas of study into effective public health interventions—the fact that most animal models of disease lack predictive value for humans [147]. Shapiro supported this in terms of using mice to study emphysema [148] while Rangarajan and Weinberg reported that numerous genetic differences exist between mouse cancer and human cancers stating that there are “fundamental differences” in the pathophysiology of cancer [149]. Weinberg was also quoted in *Fortune* magazine as saying animal models of cancer have little predictive value for humans [150]. Lindl et al. studied animal experiments performed in Germany from 1991 to 1993 and found that every hypothesis that came from animal models during this time failed in humans or was not tested [132].

We note for the record that models are successfully used in science on a daily basis and that animals can be used for many purposes in science and research. One of the authors has discussed this in previous publications [46-48,151]. Animal models may effectively be used as heuristics, as a source of tissues for humans, and for discovering more about life in general. However these specific uses are not the topic of this paper.

### The implications

In order to ascertain whether animal models are predictive for humans, one must analyze all the available data or at least enough to make a scientifically valid conclusion, rather than simply cherry picking supportive instances. Citing instances in which animals and humans responded similarly to a perturbation and concluding that animals are therefore useful in predicting outcomes from drugs or diseases is an example of the *fallacy of insufficient statistics*, the *proof by example* fallacy, or the *base rate fallacy*. Such fallacious reasoning results in the data being inaccurately presented, and false conclusions are inevitable. The preceding sections, as well as other data and articles, demonstrate unequivocally that animals cannot predict human response to drugs and disease.

Claims that “either we test on animals or we test on people” are similarly fallacious, presenting a *false dilemma* or *false dichotomy*. In reality, there are degrees of human testing and all require informed consent and must pass ethics committees. Human-based research and testing is not *ipso facto* unethical and in fact occurs every day. Some of the drug testing that is performed on animals is currently being done with humans in the form of microdosing, which is the administration of miniscule and non-dangerous doses of a substance to observe how it behaves in a human body [152-154]. One method of ensuring a safe starting dose for microdosing, since data from animal models is not sufficient, would be to begin administration in the picogram to nanogram (ng) range and increase appropriately. Even known toxic substances have a non-toxic dosage to serve as a starting point. For example, botulinum toxin is the most neurotoxic substance known and is toxic to adult humans in doses around 50-100 ng.

Current human-based testing also involves humans who have agreed to test new drugs. Ironically, as animal studies are not predictive for humans, the first clinical trials of a new drug in humans are themselves the most risky form of human-based research being performed in a large-scale manner today. Citing the results from animal studies in order to calm the fears of the human test subjects is unethical. Other examples of ethical human-based research and testing include observational studies such as those performed in the field of epidemiology, traditional clinical research in which two treatments are compared in three or more groups of patients. Human tissues are also used for testing and research. Human DNA is currently being studied in order to match genes with drug effects. Human experimentation in Nazi Germany is but one type of human-based research and is the exception to an otherwise ethical rule that protects humans from being harmed and/or being tested on without consent. The false dichotomy between human- and animal-based research equates all human-based research with that which occurred in Nazi Germany. This causes even the most ethical and promising of human-based research to appear as if it were unethical, and its proponents anti-human. In fact, the opposite is true.

A fundamental principle in research ethics is that research subjects must give their informed consent to participate in a study. In other words, they must freely volunteer. We need to briefly address the use of the word *volunteer* in the context of human-based research. To put it bluntly, it is completely disingenuous. Although the government and ethics committees require that people *volunteer* for a study, they are generally compensated. The dominant narrative to explain this practice is that society is not paying people to test drugs but is instead “reimbursing” them for their time and trouble. However, the element of compensation voids any possibility that consent is freely given in the true sense: research participants volunteer, for the most part, for the money. In fact, there are people who participate in studies as a full-time job. The truth is that society allows people to take risks for money [155].

Although written with the best of intentions and based on the science of the era, the Nuremberg Code set in motion a series of events that has resulted, ironically, in current research being conducted in an unethical manner. The results from animal-based research have been shown empirically to be invalid for predicting human response and this is supported and explained by theory from evolution and complexity science. Mandating animal-based research and testing is not only unnecessary, but also results in scientists being misled about important aspects of human pathophysiology. The mandate of animal-based research and testing has resulted in human harm in the following ways:

1. Directly, in the form of an assumption of safety when in fact the drug or procedure is harmful to humans.

2. Indirectly, when the drug or procedure would have been of benefit to humans but was withheld because of adverse reactions or lack of efficacy in animals.

3. Indirectly, by misleading scientists into pursuing lines of research that proved futile and/or harmful to humans. This is especially pertinent in light of the fact that human-based research would not have been misleading and would in fact have been informative.

4. Indirectly, in the form of consuming resources, such as scientists and funding, that could have been used in productive areas of research [156,157].

Moreover, the perpetuation of the myth that animal models ensure safety of drugs and procedures has led to society allowing the use of sentient animals in research when they would not otherwise have done so [48,158-161]. Giles writing in *Nature* states:

In the contentious world of animal research, one question surfaces time and again: how useful are animal experiments as a way to prepare for trials of medical treatments in humans? The issue is crucial, as public opinion is behind animal research *only if it helps develop better drugs*. Consequently, scientists defending animal experiments insist they are essential for safe clinical trials, whereas animal-rights activists vehemently maintain that they are useless [162]. (Emphasis added.)

There are ethical implications any time society is being misled, and in particular when it is being done in order to continue a process in which large sums of money are involved [48].

There are other financial implications. Scientists are choosing to conduct research using animals because grants are easier to obtain and the research is overall easier than conducting human-based research [156]. Moreover, scientists with a history of funding are the ones who eventually sit on funding committees. As long as this situation persists, the process will continue to be self-perpetuating. When animal-based research is analyzed in light of the fact that living organisms are complex systems with different evolutionary trajectories, there will be major changes in the funding of biomedical research. Given the fact that this is an enterprise that consumes over one-hundred billion dollars annually just in the US, there is, unsurprisingly, resistance to change from parties with a vested interest in the status quo [163].

The financial implications are also closely tied to another aspect of the ethical implications. The research funding pie is finite. Every dollar spent on research using animal models is a dollar that does not go to human-based research, the basic sciences of chemistry and physics, or to engineering. These are the areas that have been most productive for discovering new treatments and other interventions [157]. Since there is such a broad discrepancy between the efficacy of human-based research and basic research in terms of advancing medical care [157,164,165], funding research that uses animal models is actually unethical in respect of humans.

The legal ramifications of the science discussed above are already being manifest. Courts are experts at assessing the value of evidence and its ability to demonstrate truth and causation. For example, in *Daubert v. Merrell Dow Pharmaceuticals*[166], the US Supreme Court ruled that judges have the discretion to preclude animal-based research from being admitted when better evidence—in this case, epidemiological evidence—is available. Many US courts have ruled that since animal tests are not predictive for humans they cannot be admitted as evidence [166-170].

In discussions of this nature, invariably the question is raised: “What do you propose as an alternative” There are two points to be made in addressing this question. First, animal models simply fail as predictive modalities to the standards of medical science regardless of what else is available. This is not a unique situation in medical science. Many tests and interventions have been tested, found inadequate, and therefore discarded even though better options were not available. There is a reason *primum non nocere* is frequently cited in medicine. Second, progress is being made in finding tests that are predictive for patients. There is essentially universal agreement that predictive technologies will be human-based. This can be in the form of gene-based testing vis-à-vis microarrays, in silico testing [171] based on structure-activity relationships [172], quantitative structure-activity relationship (QSAR) [173], and using human stem cell [174,175], among other options [176] and combinations of options. Testing of intact living humans is currently in use in the form of microdosing and offers the possibility for expansion for other types of testing. Regardless of how predictive testing develops, it will in all likelihood be human-based [110,177-180].

Many scientists have attempted for years to reduce the number of animals used in testing and refine the testing methods in order to make the procedures less painful. While our discussion does not directly relate to this topic, it should be acknowledged that many in the scientific community have been involved in areas related to animal welfare.

### Summary

The Nuremberg Code was written in an era when it appeared to scientists that the similarities among mammalian species outweighed the differences. Today, in part due to advances in evolutionary biology and complexity theory, science has more knowledge about inter- and intra-species differences, and this knowledge falsifies the premises upon which the Nuremberg Code was based. Empirical evidence supports this. The only ethical option for society, and indeed the one most valuable for future medical advancement, is to replace animal-based research with modalities that demonstrate promise for drug development and disease treatment. Accordingly, policies and procedures that institutionalize animal-based research should be reformed.

## Competing interests

The authors declare that they have no competing interests.

## Authors’ contributions

Each author contributed equally to the manuscript.

## Authors’ information

Ray Greek, MD is board certified in anesthesiology, has been on staff at the University of Wisconsin and Thomas Jefferson University, and is the president and co-founder of the not-for-profit Americans For Medical Advancement ( http://www.AFMA-curedisease.org).

Annalea Pippus, JD completed law school at the University of Toronto in 2011 and is on the board of Americans For Medical Advancement.

Lawrence A. Hansen, MD is Professor of Neuroscience and Pathology, Department of Neurosciences at the University of California, San Diego.

## Pre-publication history

The pre-publication history for this paper can be accessed here:

http://www.biomedcentral.com/1472-6939/13/16/prepub

## References

[B1] Maehle A-H Trohler U Rupke N Vivisection In Historical Perspective. edn Animal Experimentation from Antiquity to the End of the Eighteenth Century: Attitudes and Arguments 1987 Croom Helm, London 14 47

[B2] Elliot P Rupke N Vivisection in Historical Perspective. edn Vivisection and the Emergence of Experimental Medicine in Nineteenth Century France 1987 Croom Helm, New York 48 77

[B3] Wax PM Elixirs, diluents, and the passage of the 1938 Federal Food, Drug and Cosmetic Act Ann Intern Med 1995 122 6 456 461 7856995 10.7326/0003-4819-122-6-199503150-00009

[B4] Nuremberg Trial United States v. Karl Brandt et al., "The Medical Case, Trials of War Criminals before the Nuremberg Military Tribunals under Control Council Law No. 10" 1949 U.S. Government Printing Office, Washington, D.C

[B5] Taylor T The Anatomy of the Nuremberg Trials: A Personal Memoir 1992 Knopf, New York

[B6] Annas GJ Grodin MA The Nazi Doctors and the Nuremberg Code 1992 Oxford University Press, Inc., New York

[B7] Harvard Law School Library Nuremberg Trials Project. A Digital Document Collection Introduction to NMT Case 1, U.S.A. v. Karl Brandt et al [ http://nuremberg.law.harvard.edu/php/docs_swi.php?DI=1&text=medical - persons]

[B8] American Medical Association American Medical Association, Board of Trustees Minutes of the May 1946 meeting, (ACHRE No. IND-072595-A), 156–157 1946 AMA Archive, Chicago, Illinois

[B9] Moreno JD Reassessing the Influence of the Nuremberg Code on American Medical Ethics Journal of Contemporary Health Law and Policy 1997 13 2 347 360 9212522

[B10] American Medical Association American Medical Association, Board of Trustees: 1946 Minutes of the 19 September 1946 meeting, AMA Archive, Chicago, Illinois (ACHRE No. IND-072595-A), 51–52 1946 AMA Archives, Chicago, IL

[B11] Ivy AC Report on War Crimes of a Medical Nature Committed in Germany and Elsewhere on German Nationals and the Nationals of Occupied Countries by the Nazi Regime during World War II," 1946. This report was not published, but it is available at the National Library of Medicine. A copy also exists in the AMA Archive (ACHRE No. DOD-063094-A) 1946

[B12] Chapter 2: The American Expert, the American Medical Association, and the Nuremburg Medical Trial [ http://www.hss.energy.gov/HealthSafety/ohre/roadmap/achre/chap2_2.html]

[B13] WMA Declaration of Helsinki Ethical Principles for Medical Research Involving Human Subjects [ http://www.wma.net/en/30publications/10policies/b3/] 19886379

[B14] LaFrance AB Bioethics and Animal Experimentation Animal Law 1996 2 157

[B15] NIH Regulations and Ethical Guidelines. Reprinted from Trials of War Criminals before the Nuremberg Military Tribunals under Control Council Law No. 10, Vol. 2 1949 U.S. Government Printing Office, Washington, D.C 181 182 [ http://ohsr.od.nih.gov/guidelines/nuremberg.html]

[B16] The Law Pertaining to the Protection of Animals JAMA 1934 102 7 551 552

[B17] New Regulations Concerning Vivisection JAMA 1933 101 14 1087 1088

[B18] Fritzsche U Animal experimentation in Nazi Germany Hosp Pract (Off Ed) 1990 16 4A 18 2135720

[B19] Fritzsche U Nazis and animal protection Anthrozoös 1992 5 4 218 219 10.2752/089279392787011296 11654070

[B20] Kenny MG A darker shade of green: medical botany, homeopathy, and cultural politics in interwar Germany Soc Hist Med 2002 15 3 481 504 10.1093/shm/15.3.481 12659098

[B21] Orlowski V Promising Protection Through Internationally Derived Duties Cornell International Law Journal 2004 36 381

[B22] Food and Drugs Act (R.S.C., 1985, c. F-27) [ http://laws.justice.gc.ca/eng/acts/F-27/page-7.html - h-12]

[B23] Food and Drug Regulations (C.R.C., c. 870) [ http://laws-lois.justice.gc.ca/eng/regulations/C.R.C.,_c._870/page-296.html?term=regulations+drugs+food+drug - s-G.01.001]

[B24] Statutory Instruments Act (R.S.C., 1985, c. S-22) [ http://laws-lois.justice.gc.ca/eng/acts/S%2D22/]

[B25] Table IV: Number of Animals Used in 2009 by Participants in the CCAC Program according to Purpose of Animal Use and the Category of Invasiveness http://www.ccac.ca/en_/publications/audf/stats-aud/table-IV/2009]

[B26] US Food and Drug Administration International Conference on Harmonisation; Draft Guidance on M3(R2) Nonclinical Safety Studies for the Conduct of Human Clinical Trials and Marketing Authorization for Pharmaceuticals Fed Regist 73 ed. HHSs 51491 2 [ http://www.fda.gov/cder/guidance/8500dft.htm] 20349552

[B27] Paul JR A History of Poliomyelitis 1971 Yale University Press, New Haven

[B28] Ehrlich P Hata S Die experimentalle Chemotherapie der Spirillosen 1910 Springer, Berlin

[B29] Mayr E What evolution Is Basic Books 2002

[B30] LaFollette H Shanks N Animal Experimentation: The Legacy of Claude Bernard Int Stud Philos Sci 1994 8 3 195 210 10.1080/02698599408573495

[B31] Bernard C An Introduction to the Study of Experimental Medicine 1957 Dover, New York (1865)

[B32] Cheung DS Warman ML Mulliken JB Hemangioma in twins Ann Plast Surg 1997 38 3 269 274 10.1097/00000637-199703000-00014 9088466

[B33] Couzin J Cancer research. Probing the roots of race and cancer Science 2007 315 5812 592 594 10.1126/science.315.5812.592 17272699

[B34] Gregor Z Joffe L Senile macular changes in the black African Br J Ophthalmol 1978 62 8 547 550 10.1136/bjo.62.8.547 687553 PMC1043282

[B35] Haiman CA Stram DO Wilkens LR Pike MC Kolonel LN Henderson BE Le Marchand L Ethnic and racial differences in the smoking-related risk of lung cancer N Engl J Med 2006 354 4 333 342 10.1056/NEJMoa033250 16436765

[B36] Spielman RS Bastone LA Burdick JT Morley M Ewens WJ Cheung VG Common genetic variants account for differences in gene expression among ethnic groups Nat Genet 2007 39 2 226 231 10.1038/ng1955 17206142 PMC3005333

[B37] Stamer UM Stuber F The pharmacogenetics of analgesia Expert Opin Pharmacother 2007 8 14 2235 2245 10.1517/14656566.8.14.2235 17927480

[B38] Wilke RA Dolan ME Genetics and variable drug response JAMA: The Journal of the American Medical Association 2011 306 3 306 307 10.1001/jama.2011.998 21771992 PMC3539154

[B39] Holden C Sex and the suffering brain Science 2005 308 5728 1574 10.1126/science.308.5728.1574 15947170

[B40] Kaiser J Gender in the pharmacy: does it matter? Science 2005 308 5728 1572 10.1126/science.308.5728.1572 15947169

[B41] Simon V Wanted: women in clinical trials Science 2005 308 5728 1517 10.1126/science.1115616 15947140

[B42] Wald C Wu C Of Mice and Women: The Bias in Animal Models Science 2010 327 5973 1571 1572 10.1126/science.327.5973.1571 20339045

[B43] Willyard C HIV gender clues emerge Nat Med 2009 15 8 830 19661976 10.1038/nm0809-830b

[B44] LaFollette H Shanks N Animal models in biomedical research: some epistemological worries Public Aff Q 1993 7 2 113 130 11652915

[B45] LaFollette H Shanks N Brute Science: Dilemmas of animal experimentation 1996 Routledge, London and New York

[B46] Shanks N Greek R Animal Models in Light of Evolution 2009 Brown Walker, Boca Raton

[B47] Shanks N Greek R Greek J Are animal models predictive for humans? Philos Ethics Humanit Med 2009 4 1 2 10.1186/1747-5341-4-2 19146696 PMC2642860

[B48] Greek R Greek J Is the use of sentient animals in basic research justifiable? Philos Ethics Humanit Med 2010 5 14 10.1186/1747-5341-5-14 20825676 PMC2949619

[B49] Greek R Shanks N Rice MJ The History and Implications of Testing Thalidomide on Animals The Journal of Philosophy, Science & Law 2011 11

[B50] Lewis EB A gene complex controlling segmentation in Drosophila Nature 1978 276 5688 565 570 10.1038/276565a0 103000

[B51] McGinnis W Hart CP Gehring WJ Ruddle FH Molecular cloning and chromosome mapping of a mouse DNA sequence homologous to homeotic genes of Drosophila Cell 1984 38 3 675 680 10.1016/0092-8674(84)90262-9 6091896

[B52] Gellon G McGinnis W Shaping animal body plans in development and evolution by modulation of Hox expression patterns Bioessays 1998 20 2 116 125 10.1002/(SICI)1521-1878(199802)20:2<116::AID-BIES4>3.0.CO;2-R 9631657

[B53] Slack JM Holland PW Graham CF The zootype and the phylotypic stage Nature 1993 361 6412 490 492 10.1038/361490a0 8094230

[B54] McPherson JD Marra M Hillier L Waterston RH Chinwalla A Wallis J Sekhon M Wylie K Mardis ER Wilson RK A physical map of the human genome Nature 2001 409 6822 934 941 10.1038/35057157 11237014

[B55] Venter JC Adams MD Myers EW Li PW Mural RJ Sutton GG Smith HO Yandell M Evans CA Holt RA The sequence of the human genome Science 2001 291 5507 1304 1351 10.1126/science.1058040 11181995

[B56] King MC Wilson AC Evolution at two levels in humans and chimpanzees Science 1975 188 4184 107 116 10.1126/science.1090005 1090005

[B57] Caccia S Garattini S Pasina L Nobili A Predicting the clinical relevance of drug interactions from pre-approval studies Drug Saf 2009 32 11 1017 1039 10.2165/11316630-000000000-00000 19810775

[B58] Martignoni M Groothuis GM de Kanter R Species differences between mouse, rat, dog, monkey and human CYP-mediated drug metabolism, inhibition and induction Expert Opin Drug Metab Toxicol 2006 2 6 875 894 10.1517/17425255.2.6.875 17125407

[B59] Donato MT Castell JV Strategies and molecular probes to investigate the role of cytochrome P450 in drug metabolism: focus on in vitro studies Clin Pharmacokinet 2003 42 2 153 178 10.2165/00003088-200342020-00004 12537515

[B60] Gonzalez-Angulo AM Hortobagyi GN Esteva FJ Adjuvant Therapy with Trastuzumab for HER-2/neu-Positive Breast Cancer Oncologist 2006 11 8 857 867 10.1634/theoncologist.11-8-857 16951389

[B61] Wagner GP Amemiya C Ruddle F Hox cluster duplications and the opportunity for evolutionary novelties Proc Natl Acad Sci U S A 2003 100 25 14603 14606 10.1073/pnas.2536656100 14638945 PMC299744

[B62] Amores A Force A Yan YL Joly L Amemiya C Fritz A Ho RK Langeland J Prince V Wang YL Zebrafish hox clusters and vertebrate genome evolution Science 1998 282 5394 1711 1714 9831563 10.1126/science.282.5394.1711

[B63] Garcia-Fernandez J Hox, ParaHox, ProtoHox: facts and guesses Heredity 2005 94 2 145 152 10.1038/sj.hdy.6800621 15578045

[B64] Paracelsus Der Buecher und Schriften VII 1590 172

[B65] Goldstein BD Henifin MS Kassirer JP, Kessler G Reference Manual on Scientific Evidence Reference Guide on Toxicology 2011 Third Edition National Academy of Sciences, Washington DC 633 685

[B66] Roses AD Pharmacogenetics and the practice of medicine Nature 2000 405 6788 857 865 10.1038/35015728 10866212

[B67] Bruder CE Piotrowski A Gijsbers AA Andersson R Erickson S de Stahl TD Menzel U Sandgren J von Tell D Poplawski A Phenotypically concordant and discordant monozygotic twins display different DNA copy-number-variation profiles Am J Hum Genet 2008 82 3 763 771 10.1016/j.ajhg.2007.12.011 18304490 PMC2427204

[B68] Fraga MF Ballestar E Paz MF Ropero S Setien F Ballestar ML Heine-Suner D Cigudosa JC Urioste M Benitez J Epigenetic differences arise during the lifetime of monozygotic twins Proc Natl Acad Sci U S A 2005 102 30 10604 10609 10.1073/pnas.0500398102 16009939 PMC1174919

[B69] Javierre BM Fernandez AF Richter J Al-Shahrour F Martin-Subero JI Rodriguez-Ubreva J Berdasco M Fraga MF O'Hanlon TP Rider LG Changes in the pattern of DNA methylation associate with twin discordance in systemic lupus erythematosus Genome Res 2010 20 2 170 179 10.1101/gr.100289.109 20028698 PMC2813473

[B70] Wong AH Gottesman II Petronis A Phenotypic differences in genetically identical organisms: the epigenetic perspective Hum Mol Genet 2005 14 1 R11 18 10.1093/hmg/ddi116 15809262

[B71] Blair E Predictive tests and personalised medicine Drug Discovery World 2009 10 4 27 31

[B72] Dolgin E Big pharma moves from 'blockbusters' to 'niche busters' Nat Med 2010 16 8 837 837 10.1038/nm0810-837a 20689537

[B73] Flaherty KT Puzanov I Kim KB Ribas A McArthur GA Sosman JA O'Dwyer PJ Lee RJ Grippo JF Nolop K Inhibition of mutated, activated BRAF in metastatic melanoma N Engl J Med 2010 363 9 809 819 10.1056/NEJMoa1002011 20818844 PMC3724529

[B74] Hudson KL Genomics, health care, and society N Engl J Med 2011 365 11 1033 1041 10.1056/NEJMra1010517 21916641

[B75] Hughes AR Spreen WR Mosteller M Warren LL Lai EH Brothers CH Cox C Nelsen AJ Hughes S Thorborn DE Pharmacogenetics of hypersensitivity to abacavir: from PGx hypothesis to confirmation to clinical utility Pharmacogenomics J 2008 8 6 365 374 10.1038/tpj.2008.3 18332899

[B76] Serrano D Lazzeroni M Zambon CF Macis D Maisonneuve P Johansson H Guerrieri-Gonzaga A Plebani M Basso D Gjerde J Efficacy of tamoxifen based on cytochrome P450 2D6, CYP2C19 and SULT1A1 genotype in the Italian Tamoxifen Prevention Trial Pharmacogenomics J 2011 11 2 100 107 10.1038/tpj.2010.17 20309015

[B77] Wang D Guo Y Wrighton SA Cooke GE Sadee W Intronic polymorphism in CYP3A4 affects hepatic expression and response to statin drugs Pharmacogenomics J 2011 11 4 274 86 10.1038/tpj.2010.28 20386561 PMC3248744

[B78] Lyman GH Cosler LE Kuderer NM Hornberger J Impact of a 21-gene RT-PCR assay on treatment decisions in early-stage breast cancer: an economic analysis based on prognostic and predictive validation studies Cancer 2007 109 6 1011 1018 10.1002/cncr.22506 17311307

[B79] Smalley KS Sondak VK Melanoma–an unlikely poster child for personalized cancer therapy N Engl J Med 2010 363 9 876 878 10.1056/NEJMe1005370 20818849

[B80] Thomas H Cancer Treatments get Personal New Scientist 2009 2704 48 50

[B81] Weiss ST McLeod HL Flockhart DA Dolan ME Benowitz NL Johnson JA Ratain MJ Giacomini KM Creating and evaluating genetic tests predictive of drug response Nat Rev Drug Discov 2008 7 7 568 574 10.1038/nrd2520 18587383 PMC2682785

[B82] Gad S Gad S Animal Models in Toxicology Preface 2007 CRC Press, Boca Rotan 1 18

[B83] Hau J Hau J, Hoosier GK Jr Handbook of Laboratory Animal Science Second Edition Animal Models. Volume II Animal Models 2003 2 CRC Press, Boca Rotan 1 9

[B84] Longer Tests on Lab Animals Urged for Potential Carcinogens [ http://www.cspinet.org/new/200811172.html]

[B85] Huff J Jacobson MF Davis DL The limits of two-year bioassay exposure regimens for identifying chemical carcinogens Environ Health Perspect 2008 116 11 1439 1442 10.1289/ehp.10716 19057693 PMC2592260

[B86] Litchfield JT Jr Symposium on clinical drug evaluation and human pharmacology. XVI. Evaluation of the safety of new drugs by means of tests in animals Clin Pharmacol Ther 1962 3 665 672 14465857 10.1002/cpt196235665

[B87] Collins JM McDevitt DG Shanks RG Swanton JG The cardio-toxicity of isoprenaline during hypoxia Br J Pharmacol 1969 36 1 35 45 10.1111/j.1476-5381.1969.tb08301.x 5768127 PMC1703542

[B88] Inman WH Monitoring for Drug Safety 1980

[B89] Stolley PD Asthma mortality. Why the United States was spared an epidemic of deaths due to asthma. Am Rev Respir Dis 1972 105 6 883 890 5032708 10.1164/arrd.1972.105.6.883

[B90] Stolley PD Schinnar R Fatal asthma Lancet 1979 2 8148 897 90979 10.1016/s0140-6736(79)92702-8

[B91] Rosenblum I Wohl A Stein AA Studies in Cardiac Necrosis. 3. Metabolic Effects of Sympathomimetic Amines Producing Cardiac Lesions Toxicol Appl Pharmacol 1965 7 344 351 10.1016/0041-008X(65)90103-1 14298025

[B92] Rosenblum I Wohl A Stein AA Studies in Cardiac Necrosis. Ii. Cardiovascular Effects of Sympathomimetic Amines Producing Cardiac Lesions Toxicol Appl Pharmacol 1965 7 9 17 10.1016/0041-008X(65)90068-2 14256610

[B93] Rosenblum I Wohl A Stein AA Studies in Cardiac Necrosis. I. Production of Cardiac Lesions with Sympathomimetic Amines Toxicol Appl Pharmacol 1965 7 1 8 14256598 10.1016/0041-008x(65)90067-0

[B94] Fletcher AP Drug safety tests and subsequent clinical experience J R Soc Med 1978 71 9 693 696 712750 10.1177/014107687807100915 PMC1436259

[B95] Heywood R Target organ toxicity Toxicol Lett 1981 8 6 349 358 10.1016/0378-4274(81)90125-9 7302965

[B96] Heywood R Target organ toxicity II Toxicol Lett 1983 18 1–2 83 88 6623552 10.1016/0378-4274(83)90075-9

[B97] Salsburg D The lifetime feeding study in mice and rats–an examination of its validity as a bioassay for human carcinogens Fundam Appl Toxicol 1983 3 1 63 67 10.1016/S0272-0590(83)80174-2 6884625

[B98] Garattini S Toxic effects of chemicals: difficulties in extrapolating data from animals to man Crit Rev Toxicol 1985 16 1 1 29 10.3109/10408448509041323 3910353

[B99] Heywood R Lumley CE, Walker S Animal Toxicity Studies: Their Relevance for Man Clinical Toxicity--Could it have been predicted? Post-marketing experience 1990 Quay, Lancaster 57 67

[B100] Olson H Betton G Robinson D Thomas K Monro A Kolaja G Lilly P Sanders J Sipes G Bracken W Concordance of the toxicity of pharmaceuticals in humans and in animals Regul Toxicol Pharmacol 2000 32 1 56 67 10.1006/rtph.2000.1399 11029269

[B101] Suter K Lumley C, Walker S Animal Toxicity Studies: Their Relevance for Man What can be learned from case studies? The company approach 1990 Quay, Lancaster 71 78

[B102] Lumley C Lumley C, Walker S Animal Toxicity Studies: Their Relevance for Man Clinical toxicity: could it have been predicted? Premarketing experience 1990 Quay, London 49 56

[B103] Spriet-Pourra C Auriche M SCRIP Reports 1994 PJB

[B104] Eason CT Bonner FW Parke DV The importance of pharmacokinetic and receptor studies in drug safety evaluation Regul Toxicol Pharmacol 1990 11 3 288 307 10.1016/0273-2300(90)90028-A 2196638

[B105] Igarashi T Parkinson NM C, Lumley C, Walker SR CMR Workshop: The Timing of Toxicological Studies to Support Clinical Trials The duration of toxicity studies required to support repeated dosing in clinical investigation—A toxicologists opinion 1994 Kluwer, Boston/UK 67 74

[B106] Igarashi T Nakane S Kitagawa T Predictability of clinical adverse reactions of drugs by general pharmacology studies J Toxicol Sci 1995 20 2 77 92 10.2131/jts.20.77 7473897

[B107] Igarashi T Yabe T Noda K Study design and statistical analysis of toxicokinetics: a report of JPMA investigation of case studies J Toxicol Sci 1996 21 5 497 504 10.2131/jts.21.5_497 9035061

[B108] Weaver JL Staten D Swann J Armstrong G Bates M Hastings KL Detection of systemic hypersensitivity to drugs using standard guinea pig assays Toxicology 2003 193 3 203 217 10.1016/S0300-483X(03)00267-1 14599760

[B109] Willis RC The Virtual Patient Modern Drug Discovery 2003 6 2 35 40

[B110] Sankar U The Delicate Toxicity Balance in Drug Discovery Scientist 2005 19 15 32

[B111] Lin JH Species similarities and differences in pharmacokinetics Drug Metab Dispos 1995 23 10 1008 1021 8654187

[B112] Ekwall B Overview of the Final MEIC Results: II. The In Vitro--In Vivo Evaluation, Including the Selection of a Practical Battery of Cell Tests for Prediction of Acute Lethal Blood Concentrations in Humans Toxicol In Vitro 1999 13 4–5 665 673 20654532 10.1016/s0887-2333(99)00061-2

[B113] Roggeband R York M Pericoi M Braun W Eye irritation responses in rabbit and man after single applications of equal volumes of undiluted model liquid detergent products Food and chemical toxicology : an international journal published for the British Industrial Biological Research Association 2000 38 8 727 734 10.1016/S0278-6915(00)00057-0 10908820

[B114] Miller M Bhalla K An urgent need to restrict access to pesticides based on human lethality PLoS Med 2010 7 10 e1000358 10.1371/journal.pmed.1000358 21048989 PMC2964339

[B115] Dawson AH Eddleston M Senarathna L Mohamed F Gawarammana I Bowe SJ Manuweera G Buckley NA Acute human lethal toxicity of agricultural pesticides: a prospective cohort study PLoS Med 2010 7 10 e1000357 10.1371/journal.pmed.1000357 21048990 PMC2964340

[B116] Sietsema WK The absolute oral bioavailability of selected drugs Int J Clin Pharmacol Ther Toxicol 1989 27 4 179 211 2654032

[B117] Giri S Bader A Foundation review: Improved preclinical safety assessment using micro-BAL devices: the potential impact on human discovery and drug attrition Drug Discovery Today 2011 16 9/10 382 397 21354326 10.1016/j.drudis.2011.02.012

[B118] Browne LJ Taylor LL Predictive chemoinformatics: applications to the pharmaceutical industry Drug Discovery World 2002 71 77 Fall

[B119] Gura T Cancer Models: Systems for identifying new drugs are often faulty Science 1997 278 5340 1041 1042 10.1126/science.278.5340.1041 9381203

[B120] Wang L McLeod HL Weinshilboum RM Genomics and drug response N Engl J Med 2011 364 12 1144 1153 10.1056/NEJMra1010600 21428770 PMC3184612

[B121] Mann R Andrews E Pharmacovigilance 2006 2 John Wiley and Sons, Chichester

[B122] Kaplowitz N Idiosyncratic drug hepatotoxicity Nat Rev Drug Discov 2005 4 6 489 499 10.1038/nrd1750 15931258

[B123] Fourches D Barnes JC Day NC Bradley P Reed JZ Tropsha A Cheminformatics analysis of assertions mined from literature that describe drug-induced liver injury in different species Chem Res Toxicol 2010 23 1 171 183 10.1021/tx900326k 20014752 PMC2850112

[B124] Koppanyi T Avery MA Species differences and the clinical trial of new drugs: a review Clin Pharmacol Ther 1966 7 2 250 270 5327179 10.1002/cpt196672250

[B125] Collins JM Inter-species differences in drug properties Chem Biol Interact 2001 134 3 237 242 10.1016/S0009-2797(01)00158-2 11336972

[B126] Knight A Bailey J Balcombe J Animal carcinogenicity studies: 1. Poor human predictivity Altern Lab Anim 2006 34 1 19 27 16522147 10.1177/026119290603400117

[B127] Oser BL The rat as a model for human toxicological evaluation J Toxicol Environ Health 1981 8 4 521 542 10.1080/15287398109530089 7338928

[B128] Calabrese EJ Suitability of animal models for predictive toxicology: theoretical and practical considerations Drug Metab Rev 1984 15 3 505 523 10.3109/03602538409029971 6435984

[B129] Calabrese EJ Principles of Animal Extrapolation 1991 CRC Press, Boca Rotan

[B130] Perel P Roberts I Sena E Wheble P Briscoe C Sandercock P Macleod M Mignini LE Jayaram P Khan KS Comparison of treatment effects between animal experiments and clinical trials: systematic review BMJ 2007 334 7586 197 10.1136/bmj.39048.407928.BE 17175568 PMC1781970

[B131] Testing Treatment on Animals: Relevance to Humans [ http://www.pcpoh.bham.ac.uk/publichealth/methodology/docs/invitations/JH18_Final_Report_April_2006.pdf]

[B132] Lindl T Voelkel M Kolar R [Animal experiments in biomedical research. An evaluation of the clinical relevance of approved animal experimental projects] ALTEX 2005 22 3 143 151 16186990

[B133] Lindl T Völkel M Kolar R Animal experiments in biomedical research. An evaluation of the clinical relevance of approved animal experimental projects: No evident implementation in human medicine within more than 10 years. [Lecture abstract.] ALTEX 2006 23 111

[B134] Tolman KG The liver and lovastatin Am J Cardiol 2002 89 12 1374 1380 10.1016/S0002-9149(02)02355-X 12062731

[B135] Navarro VJ Senior JR Drug-related hepatotoxicity N Engl J Med 2006 354 7 731 739 10.1056/NEJMra052270 16481640

[B136] Dixit R Boelsterli U Healthy animals and animal models of human disease(s) in safety assessment of human pharmaceuticals, including therapeutic antibodies Drug Discovery Today 2007 12 7–8 336 342 17395094 10.1016/j.drudis.2007.02.018

[B137] Toxicity Testing in the 21st Century [ http://www.alttox.org/ttrc/overarching-challenges/way-forward/andersen/]

[B138] Hartung T Toxicology for the twenty-first century Nature 2009 460 7252 208 212 10.1038/460208a 19587762

[B139] Force T Kolaja KL Cardiotoxicity of kinase inhibitors: the prediction and translation of preclinical models to clinical outcomes Nat Rev Drug Discov 2011 10 2 111 126 10.1038/nrd3252 21283106

[B140] Suntharalingam G Perry MR Ward S Brett SJ Castello-Cortes A Brunner MD Panoskaltsis N Cytokine storm in a phase 1 trial of the anti-CD28 monoclonal antibody TGN1412 N Engl J Med 2006 355 10 1018 1028 10.1056/NEJMoa063842 16908486

[B141] Dayan CM Wraith DC Preparing for first-in-man studies: the challenges for translational immunology post-TGN1412 Clin Exp Immunol 2008 151 2 231 234 10.1111/j.1365-2249.2007.03559.x 18190459 PMC2276942

[B142] Chapman AR Addressing the Ethical Challenges of First-in-Human Trials J Clinic Res Bioeth 2011 2 4 113

[B143] Marshall E Gene therapy on trial Science 2000 288 5468 951 957 10.1126/science.288.5468.951 10841710

[B144] Horstmann E McCabe MS Grochow L Yamamoto S Rubinstein L Budd T Shoemaker D Emanuel EJ Grady C Risks and benefits of phase 1 oncology trials, 1991 through 2002 N Engl J Med 2005 352 9 895 904 10.1056/NEJMsa042220 15745980

[B145] Lavery JV How can institutional review boards best interpret preclinical data? PLoS medicine 2011 8 3 e1001011 10.1371/journal.pmed.1001011 21423343 PMC3050913

[B146] Anderson J Kimmelman J Extending Clinical Equipoise to Phase I Trials Involving Patients: Unresolved Problems Kennedy Inst Ethics J 2010 20 79 81 10.1353/ken.0.0307 PMC4482670 20506695

[B147] Enna SJ Williams M Defining the role of pharmacology in the emerging world of translational research Adv Pharmacol 2009 57 1 30 20230758 10.1016/S1054-3589(08)57001-3

[B148] Shapiro SD Transgenic and gene-targeted mice as models for chronic obstructive pulmonary disease Eur Respir J 2007 29 2 375 378 17264324 10.1183/09031936.00087606

[B149] Rangarajan A Weinberg RA Opinion: Comparative biology of mouse versus human cells: modelling human cancer in mice Nat Rev Cancer 2003 3 12 952 959 10.1038/nrc1235 14737125

[B150] Leaf C Why we are losing the war on cancer Fortune 2004 77 92 March 9 15069734

[B151] Greek R Shanks N FAQs About the Use of Animals in Science: A handbook for the scientifically perplexed 2009 University Press of America, Lanham

[B152] Lappin G Garner RC Big physics, small doses: the use of AMS and PET in human microdosing of development drugs Nat Rev Drug Discov 2003 2 3 233 240 10.1038/nrd1037 12612650

[B153] Lappin G Garner RC The utility of microdosing over the past 5 years Expert Opinion on Drug Metabolism & Toxicology 2008 4 12 1499 1506 10.1517/17425250802531767 19040326

[B154] Lappin G Kuhnz W Jochemsen R Kneer J Chaudhary A Oosterhuis B Drijfhout WJ Rowland M Garner RC Use of microdosing to predict pharmacokinetics at the therapeutic dose: experience with 5 drugs Clin Pharmacol Ther 2006 80 3 203 215 10.1016/j.clpt.2006.05.008 16952487

[B155] Abadie R The Professional Guinea Pig: Big Pharma and the Risky World of Human Subjects 2010 Duke University Press Books, Durham

[B156] Rice MJ The institutional review board is an impediment to human research: the result is more animal-based research Philosophy, ethics, and humanities in medicine : PEHM 2011 6 12 10.1186/1747-5341-6-12 PMC3127833 21649895

[B157] Rothwell PM Funding for practice-oriented clinical research Lancet 2006 368 9532 262 266 10.1016/S0140-6736(06)69010-7 16860680

[B158] Scientific Achievements Less Prominent Than a Decade Ago. Public praises science; scientists fault public, media [ http://people-press.org/report/528/]

[B159] Aldhous P Coghlan A Copley J Let the people speak New Scientist 1999 2187 May 22 11657970

[B160] Special Eurobarometer: Social values, Science and Technology [ http://ec.europa.eu/public_opinion/archives/ebs/ebs_225_report_en.pdf]

[B161] Four Moral Issues Sharply Divide Americans [ http://www.gallup.com/poll/137357/Four-Moral-Issues-Sharply-Divide-Americans.aspx?utm_source=alert&utm_medium=email&utm_campaign=syndication&utm_content=morelink&utm_term=Moral Issues]

[B162] Giles J Animal experiments under fire for poor design Nature 2006 444 7122 981 10.1038/444981a 17183281

[B163] U.S. Investment In Health Research 2009 [ http://www.researchamerica.org/uploads/healthdollar09.pdf]

[B164] Ioannidis JPA Contradicted and Initially Stronger Effects in Highly Cited Clinical Research JAMA: The Journal of the American Medical Association 2005 294 2 218 228 10.1001/jama.294.2.218 16014596

[B165] Crowley WF Jr Translation of basic research into useful treatments: how often does it occur? Am J Med 2003 114 6 503 505 10.1016/S0002-9343(03)00119-0 12727585

[B166] Daubert v. Merrell Dow Pharms., 509 U.S. 579, 584 (U.S., 1993) In

[B167] Viterbo v. Dow Chemical Co., 826 F.2d 420 (5th Cir. 1987) In

[B168] Selwood v. Oxford Chemicals, Inc., No. 90–1048 (M.D. Pa. June 28, 1991) In

[B169] Joiner v. General Elec. Co., 864 F. Supp. 1310, 1323 (N.D. Ga. 1994) In

[B170] Bourne v. E.I.DuPont de Nemours and Company, 189 F Supp. 2d 482 (S.D. W.Va. 2002)

[B171] Fliri AF Loging WT Thadeio PF Volkmann RA Biological spectra analysis: Linking biological activity profiles to molecular structure Proc Natl Acad Sci U S A 2005 102 2 261 266 10.1073/pnas.0407790101 15625110 PMC539313

[B172] Borman S Drugs by Design Chemical & Engineering News 2005 28

[B173] Models Predict Toxicity of Compounds [ http://www.dddmag.com/product-Models-Predict-Toxicity-of-Compounds-052611.aspx?et_cid=1799359&et_rid=45518461&linkid=http%3a%2f%2fwww.dddmag.com%2fproduct-Models-Predict-Toxicity-of-Compounds-052611.aspx]

[B174] Hoffman LM Carpenter MK Characterization and culture of human embryonic stem cells Nat Biotechnol 2005 23 6 699 708 10.1038/nbt1102 15940242

[B175] Sinha G Cell biology. Human embryonic stem cells may be toxicology's new best friends Science 2005 308 5728 1538 10.1126/science.308.5728.1538 15947151

[B176] Geerts H Of mice and men: bridging the translational disconnect in CNS drug discovery CNS Drugs 2009 23 11 915 926 10.2165/11310890-000000000-00000 19845413

[B177] Altman L Who Goes First? The Story of Self-Experimentation in Medicine 1998 University of California Press

[B178] Greek J Greek R What Will We Do if We Don't Experiment on Animals? Trafford 2004

[B179] Regenberg A Mathews DJ Blass DM Bok H Coyle JT Duggan P Faden R Finkel J Gearhart JD Hillis A The role of animal models in evaluating reasonable safety and efficacy for human trials of cell-based interventions for neurologic conditions J Cereb Blood Flow Metab 2009 29 1 1 9 10.1038/jcbfm.2008.98 18728679 PMC2682696

[B180] Littman BH Williams SA The ultimate model organism: progress in experimental medicine Nat Rev Drug Discov 2005 4 8 631 638 10.1038/nrd1800 16056389

